# Should we treat fever in critically ill patients? A summary of the current evidence from three randomized controlled trials

**DOI:** 10.1590/S1679-45082014RW2785

**Published:** 2014

**Authors:** Ary Serpa, Victor Galvão Moura Pereira, Giancarlo Colombo, Farah Christina de la Cruz Scarin, Camila Menezes Souza Pessoa, Leonardo Lima Rocha

**Affiliations:** 1 Faculdade de Medicina do ABC, Santo Andre, SP, Brazil.; 2 Hospital Israelita Albert Einstein, São Paulo, SP, Brazil.

**Keywords:** Fever/drug therapy, Critical illness, Critical care, Antipyretics/therapeutic use, Intensive care units

## Abstract

Fever is a nonspecific response to various types of infectious or non-infectious insult and its significance in disease remains an enigma. Our aim was to summarize the current evidence for the use of antipyretic therapy in critically ill patients. We performed systematic review and meta-analysis of publications from 1966 to 2013. The MEDLINE and CENTRAL databases were searched for studies on antipyresis in critically ill patients. The meta-analysis was limited to: randomized controlled trials; adult human critically ill patients; treatment with antipyretics in one arm *versus* placebo or non-treatment in another arm; and report of mortality data. The outcomes assessed were overall intensive care unit mortality, changes in temperature, intensive care unit length of stay, and hospital length of stay. Three randomized controlled trials, covering 320 participants, were included. Patients treated with antipyretic agents showed similar intensive care unit mortality (risk ratio 0.91, with 95% confidence interval 0.65-1.28) when compared with controls. The only difference observed was a greater decrease in temperature after 24 hours in patients treated with antipyretics (-1.70±0.40 *versus* - 0.56±0.25ºC; p=0.014). There is no difference in treating or not the fever in critically ill patients.

## INTRODUCTION

Fever is a nonspecific response to various types of infectious or non-infectious insults and its significance in diseases remains an enigma. Although fever is primarily a symptom of infection, it is unclear whether the fever is harmful or beneficial to the host.^(1)^ In a classical study, Kluger et al. showed that an elevation in temperature in lizards following experimental bacterial infection results in a significant increase in host survival.^(2)^ In a recent multi-centered prospective observational study, Lee et al. showed that the association between fever and mortality and of type of antipyretic treatment and mortality was different between septic and non-septic patients. In non-septic patients, temperature ≥39.5°C was associated with 28-day mortality. However, in septic patients, administration of antipyretic therapy was independently associated with increased mortality.^(1)^

The use of antipyretic therapy in febrile critically ill patients is inconsistent, and there are strong arguments both for and against it. Although pyrexia can be an adaptive response to stress, it can increase the oxygen consumption and cause discomfort to patients. Previous randomized controlled trials assessing antipyretic therapy in critically ill patients have been small, underpowered and provided divergent results. In front of these conflicting results, meta-analysis provides an useful tool to pool and analyze the data from these studies.

## OBJECTIVE

The widespread use of antipyretic methods in intensive care units’ patients is not supported by clinical data, and fever control may be harmful, particularly when an infectious disease is progressing. Since this is a controversial topic we conducted a brief systematic review and meta-analysis of the literature to summarize the current evidence for the use o antipyretic therapy in critically ill patients.

## METHODS

### Literature search and data extraction

The online database of MedLine (1966-2013) and Cochrane Register of Controlled Trials (CENTRAL) were searched for studies that fulfill the following inclusion criteria: randomized controlled trials; adult human critically ill patients; treatment with antipyretics in one arm *versus* placebo or non-treatment in other arm; and report of mortality data.

The following terms were combined in the search strategy: (acetaminophen [MeSH Terms] OR NSAID [MeSH Terms] OR aspirin [MeSH Terms] OR antipyrine [MeSH Terms] OR cooling) AND (critical illness [MeSH Terms] OR critical care [MeSH Terms] OR Intensive care [MeSH Terms]). All review articles and cross-referenced studies from retrieved articles were screened for pertinent information. When we found duplicate reports of the same study in preliminary abstracts and articles, we analyzed data from the most complete data set.

### Outcomes and data analysis

The primary outcome was overall ICU mortality in patients treated with antipyretics *versus* patients not treated. The secondary outcomes included change in temperature, ICU length of stay, and hospital length of stay. According to PICOS statement, we evaluated: *P*, critically ill patients; *I*, antipyresis; *C*, no antipyresis; *O*, ICU mortality; *S*, intensive care unit.

We extracted data regarding the study design, patient characteristics, overall survival, and mean change in body temperature. For the analysis of survival, we calculated a pooled estimate of risk ratio (RR) in the individual studies using a random effect model according to Mantel and Haenszel and graphically represented these results using forest plot graphs. For continuous variables, we used the standardized mean difference (SMD), which is the difference in means divided by a standard deviation (SD). The homogeneity assumption was checked by a χ^2^ test with a *df* equal to the number of analyzed studies minus 1. Also, the heterogeneity was measured by the I^2^, which describes the percentage of total variation across studies, that is due to heterogeneity rather than chance. I^2^ was calculated from basic results obtained from a typical meta-analysis as I^2^ = 100% x (*Q* – df)/*Q*, where *Q* is Cochran’s heterogeneity statistic and df is the degrees of freedom. A percentage of zero indicates no observed heterogeneity, and larger values show increasing heterogeneity. When heterogeneity was found we tried to identify and describe the reason.

Parametric variables were presented as the mean±SD and non-parametric variables were presented as the median (interquartile range). All analyses were conducted with Review Manager v.5.1.1 and Statistical Package for Social Sciences (SPSS) v.16.0.1. For all analyses, p values <0.05 were considered significant.

## RESULTS

**Figure 1:**
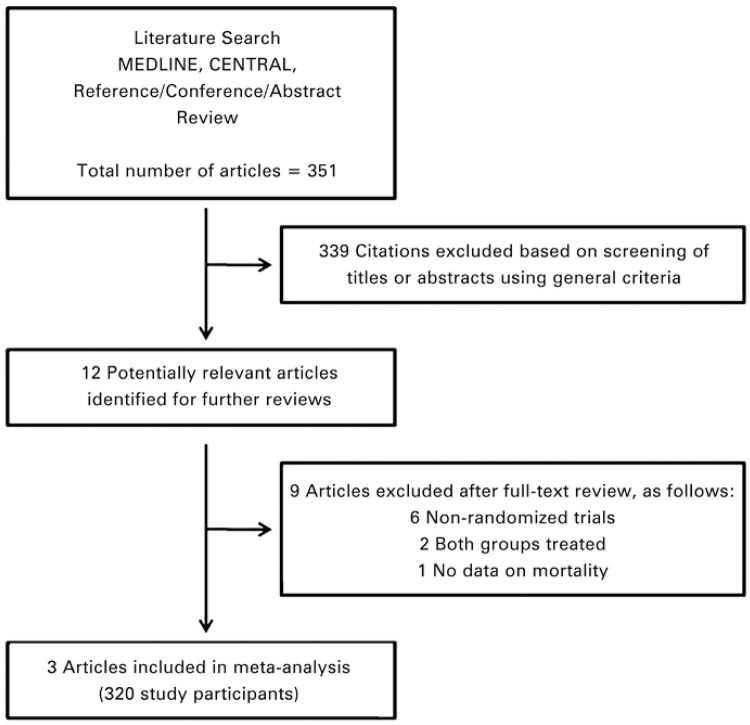
Literature search strategy

A comprehensive literature search yielded 351 references, of which 339 articles were excluded during the first screening, which was based on abstracts or titles, leaving 12 articles for full text review. During this review, nine articles were excluded for the following reasons: non-randomized trial (n=6); both groups treated (n=2); and no data about mortality (n=1). Finally, three articles (320 participants) were included in the final analysis^(3-5)^ (Figure 1 and Table 1).

**Table 1:** Scientific quality of included studies

Studies	Allocation concealment	Baseline similarity	Early stopping^*^	Lost to follow-up	Intention-to-treat analysis
Gozzoli et al.^(3)^ Jadad score: 3	Sealed envelopes	Age: similar Illness severity: similar (SAPS II)	No	No	NS
Schulman et al.^(4)^ Jadad score: 3	Sealed envelopes	Age: similar Illness severity: similar (APACHE II)	No	No	NS
Schortgen et al.^(5)^ Jadad score: 3	Telephone system	Age: similar Illness severity: similar (SAPS III)	No	No	Yes

* : Early termination for benefit or futility and the presence of an explicit a *priori* stopping rules. NS: not significant.

All three studies analyzed were randomized controlled trials and in two the treatment of fever was with an external cooling device^(3,5)^ and in the last with acetaminophen.^(4)^ In one study the patients in the control group could be treated if the temperature reached a determined value^(4)^ and in the other two no intervention was made in the control group. Two studies evaluated surgical patients^(3,4)^ and one assessed patients with septic shock at ICU stay.^(5)^ Characteristics and outcomes of the studies analyzed are exposed in table 2.

**Table 2:** Characteristics and outcomes of the studies included in the meta-analysis

Characteristics	Gozzoli et al.^(3)^		Schulman et al.^(4)^		Schortgen et al.^(5)^	
	Treatment	No treatment	Treatment	No treatment	Treatment	No treatment
Scenario	Surgical patients		Surgical patients		Septic shock	
Measurement of T	Rectal		Not defined		Core T	
Number of patients	18	20	44	38	101	99
Age, years	54±13	53±19	47±20	47±20	62	61
Severity scores	30^*^	28^*^	12.8^**^	11.4^**^	77^***^	79^***^
Initiation of antipyresis	T≥38.5 + SIRS	Never	T>38.5	T>40.0	T>38.3	Never
Type of antipyresis	External cooling	Nothing	Acetaminophen	Acetaminophen	External cooling	Nothing
Objective of antipyresis	T≤37.5	Nothing	T<38.5	T<40.0	T<37.0	Nothing
Initial T, ºC	38.9±0.3	38.8±0.5	38.3±0.8	38.3±0.7	38.8±0.8	38.9±0.7
T after 24 hours, ºC	37.6±0.5	37.7±0.6	36.6±0.6	37.7±0.5	36.7±0.6	38.1±0.5
ICU stay, days	11±13	9±10	22±30	20±14	17±14	16±17
Hospital stay, days	28±22	31±24	-	-	36±40	28±31
ICU mortality, n (%)	2 (11)	3 (15)	7 (16)	1 (3)	35 (35)	43 (43)

* : Simplified Acute Physiology Score (SAPS) II;

** : Acute Physiology and Chronic Health Evaluation II (APACHE II);

*** : SAPS III. T: temperature; SIRS: systemic inflammatory response syndrome; ICU: intensive care unit.

**Figure 2:**
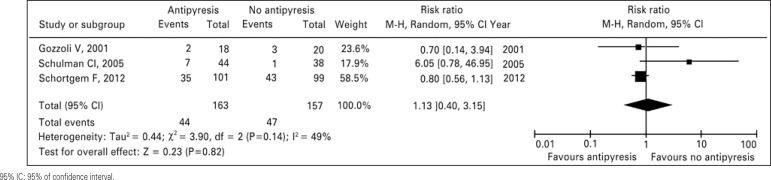
Meta-analysis of overall survival for antipyresis or no antipyresis in critically ill patients

Of 163 patients, 44 (27%) assigned to fever treatment and 47 out of 157 patients (30%) assigned as controls died during ICU stay (RR: 0.91; 95% of confidence interval – 95%CI: 0.65-1.28). There was no difference in stratified analysis between surgical patients and septic shock patients (RR: 2.19; 95%CI: 0.68-7.06; and RR: 0.80; 95%CI: 0.56-1.13, respectively). There is mild heterogeneity among the results (Table 3 and Figure 2). The visual inspection of survival analysis funnel plot revealed symmetry and the Begg test was not statistically significant (p=0.54).

**Table 3:** Characteristics and outcome of the patients analyzed in the meta-analysis

	Treatment (n=163)	Control (n=157)	p value	SMD/RR (95%CI)	Heterogeneity	p value
Age, years	54.33±7.59	53.66±7.02	0.916	-	-	-
Initial temperature, ºC	38.66±0.32	38.40±0.45	0.456	-0.06 (-0.27-0.16)	0.560	0.620
Temperature after 24 hours, ºC	36.96±0.55	37.83±0.23	0.066	-1.57 (-2.86- -0.29)	<0.0001	0.020
Change in temperature, ºC	-1.70±0.40	-0.56±0.25	0.014	-	-	-
ICU stay, days	16.66±5.50	15.00±5.56	0.731	0.08 (-0.14-0.30)	0.960	0.470
Hospital stay, days	32.00±5.65	29.50±2.12	0.618	0.17 (-0.09-0.42)	0.320	0.200
ICU mortality, number (%)	44 (27)	47 (30)	0.637	1.13 (0.40-3.15)	0.140	0.820

SMD: standardized mean difference; RR: risk ratio; 95%CI: 95% of confidence interval; ICU: intensive care unit. 95% IC: 95% of confidence interval.

**Figure 3:**
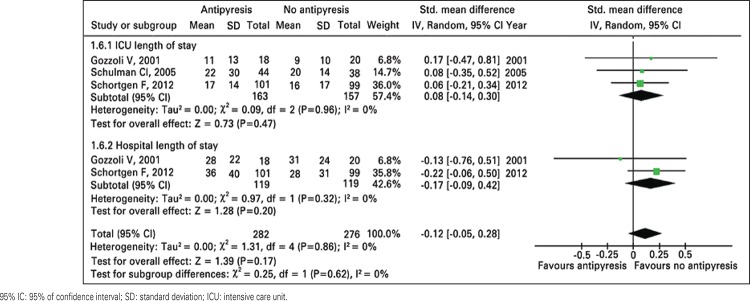
Meta-analysis of intensive care unit and hospital length of stay for antipyresis or no antipyresis in critically ill patients

**Figure 4:**
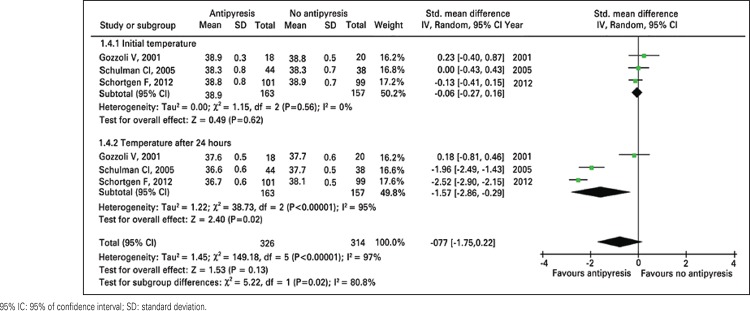
Meta-analysis of temperature at the beginning and at the end of 24 hours for antipyresis or no antipyresis in critically ill patients

**Figure 5:**
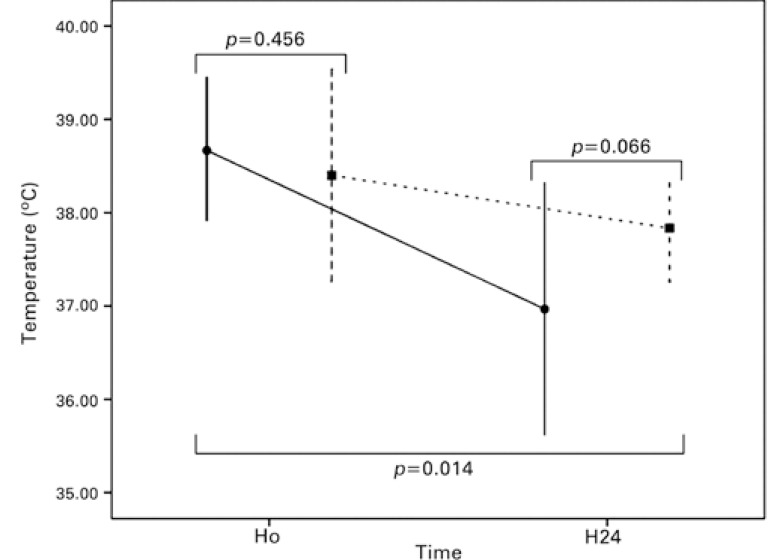
Change in temperature after 24 hours in patients treated with antipyretics (black circle and continuous line) and controls (black square and dashed line)

There is no difference in ICU and hospital length of stay between patients treated and controls (Table 2 and Figure 3). As expected, the patients treated with antipyretic agents had greater decrease in temperature during 24 hours and lower body temperature at the end of the follow-up (Table 2 and Figures 4 and 5).

## DISCUSSION

We founded evidence that the use of antipyretics for fever control in critically ill patients was not associated with better outcomes when compared to patients that were not treated. Notably, the decrease in the temperature during the first 24 hours and the temperature after 24 hours were significantly lower in the group treated with antipyresis.

Fever has been recognized as a hallmark of diseases for 4,500 to 5,000 years.^(6)^ It is due to a number of endogenous molecules able to modify the regular temperature. While the activity of pyrexin was possibly due to an endotoxin contamination, the fever-producing substance from polymorphonuclear leukocytes, and the endogenous pyrogen were candidates, now recognized as pyrogenic cytokines.^(6-9)^

In the decade of 1970, Kluger et al.^(2)^ showed that housing lizards infected with a bacteria at 42°C allowed them to survive, while all died when kept at 34°C. Thirty years after, Jiang et al.^(10)^ conducted a similar experiment in mice. Peritonitis infection was inoculated in mice housed in an ambient to allow a core temperature of 37.5 or 39.7°C. The bacterial load was exponential in the peritoneal cavity of mice with no fever and was under control in mice with fever. All mice with no fever died while 50% of those with fever survived.

Fever was found to be associated with better outcome in humans in several observational studies. In patients with *Gram*-negative bacteremia, fever was among the factors related to a decreased mortality.^(11)^ In elderly patients with community-acquired pneumonia, fever and leukocytosis were also associated with decreased mortality.^(12)^ Due to numerous experimental animal models of severe infection which antipyresis was shown to increase mortality, physician were warned about the use of antipyresis in septic patients.^(13)^

Recently, Lee et al.^(1)^ showed that, in critically ill patients, the association of fever and mortality varied according to the level of fever and it was independently associated with mortality only in subgroup ≥39.5°C of patients without sepsis. In this group of patients, it can be assumed that high fever is likely to be caused by infection and this may account for mortality. High fever is associated with cardiac arrhythmias, increased oxygen demand, brain damage, and convulsions.^(14,15) ^In patients with non-infective fever, these deleterious effects will occur without the potential benefit of fever-related protection.^(1)^

Fever is thought to inhibit the activity of viruses and bacteria and antipyretic treatment can decrease this action.^(1,16)^ Also, antipyresis in septic patients with non-steroidal anti-inflammatory drugs and acetaminophen may be toxic, as they might be associated with hypotension and renal dysfunction.^(17)^ Again, Lee et al.^(1)^ showed that mortality is higher for septic patients who fail to develop fever, supporting the argument that fever might be naturally protective. One study of trauma patients was prematurely stopped due trend toward increase in risk of infection and death in patients treated aggressively with acetaminophen and physical cooling.^(4)^ Also, two studies reported that therapy with ibuprofen in patients with sepsis did not influence mortality.^(18,19)^

Limitations of our study include the risk of bias which may exaggerate the study’s conclusion if publication is related to the strength of the results. Also, there are only three trials included, which increases the bias of these studies. We searched the references in few databases and used a simple search strategy, which could lead to loss of some studies. The analysis of physical cooling together with drugs could be another source of bias.

A large randomized controlled trial is being conducted to confirm the real effect of antipyresis in critically ill patients.^(20)^

## CONCLUSION

The results of this review suggest that antipyresis in critically ill patients was not associated with better survival compared with no treatment of the fever. Further larger studies are needed to confirm the effect of fever control on mortality and to determine whether mild hypothermia provides additional benefits in critically ill patients.
