# Supplementary material for: Should we treat fever in critically ill patients? A summary of the current evidence from three randomized controlled trials
Source: Einstein (Sao Paulo). 2014 Oct-Dec;12(4):518–23. doi: 10.1590/S1679-45082014RW2785 (PMC4879924; doi:10.1590/S1679-45082014RW2785)
Supplement: Translation

# Translation: Devemos tratar febre em doentes graves? Resumo da evidência atual de três ensaios clínicos randomizados

**Authors:** Ary Serpa, Victor Galvão Moura Pereira, Giancarlo Colombo, Farah Christina de la Cruz Scarin, Camila Menezes Souza Pessoa, Leonardo Lima Rocha

**Affiliations:** 1 Faculdade de Medicina do ABC, Santo André, SP, Brasil.; 2 Hospital Israelita Albert Einstein, São Paulo, SP, Brasil.

**Keywords:** Febre/quimioterapia, Doença grave, Cuidados críticos, Antipiréticos/uso terapêutico, Unidades de terapia intensiva

## Abstract

A febre é uma resposta não específica a vários tipos de insultos, de origem infecciosa ou não, e sua importância em doenças continua a ser um enigma. Nosso objetivo foi resumir a evidência atual para o uso de antipiréticos em pacientes graves. Foram realizadas revisão sistemática e meta-análise de publicações entre 1966 e 2013. As bases de dados MEDLINE e CENTRAL foram pesquisadas para estudos sobre antipirese em pacientes graves. A meta-análise restringiu-se a ensaios clínicos randomizados em humanos adultos; pacientes graves; tratamento com antipiréticos em um braço contra placebo ou não tratamento no outro; e dados sobre mortalidade. Os desfechos avaliados foram: mortalidade geral na unidade de terapia intensiva, mudança de temperatura e tempo de internação na unidade de terapia intensiva e no hospital. Três ensaios clínicos randomizados com 320 participantes foram incluídos. Os pacientes tratados com antipiréticos tiveram mortalidade na unidade de terapia intensiva semelhante aos controles (razão de risco de 0,91, com intervalo de confiança de 95% de 0,65-1,28). A única diferença observada foi uma diminuição na temperatura após 24 horas em pacientes tratados com antipiréticos (-1,70±0,40 x - 0,56±0,25ºC; p=0,014). Não houve diferença entre tratar ou não a febre em pacientes graves.

## INTRODUÇÃO

A febre é uma resposta inespecífica a vários tipos de insultos infecciosos ou não, e seu significado em doenças permanece um enigma. Apesar de a febre ser primariamente um sintoma de infecção, não está claro se é prejudicial ou benéfica para o hospedeiro.^(1)^ Em um estudo clássico, Kluger et al. mostraram que uma elevação na temperatura de lagartos após infecção bacteriana experimental resultou em um aumento significativo de sobrevida dos hospedeiros.^(2)^ Em um recente estudo observacional prospectivo multi-cêntrico, Lee et al. mostraram que a associação entre febre e mortalidade, e tipo de tratamento antipirético e mortalidade era diferente entre pacientes com e sem sepse. Nos pacientes não sépticos, uma temperatura ≥39,5°C estava associada a mortalidade em 28 dias. Entretanto, em pacientes sépticos, a administração de antipiréticos mostrou associação independente com maior mortalidade.^(1)^

O uso de antipiréticos em pacientes críticos febris é inconsistente e há fortes argumentos tanto a favor como contra. A febre poder ser uma resposta adaptativa ao estresse, mas pode aumentar o consumo de oxigênio e trazer desconforto ao paciente. Os ensaios randomizados controlados realizados que avaliaram o tratamento antipirético em pacientes críticos foram pequenos, carentes de poder estatístico e produziram resultados divergentes. Diante desses resultados conflitantes, uma meta-análise é uma ferramenta útil para reunir e analisar os dados desses estudos.

## OBJETIVO

O uso disseminado de métodos antipiréticos em pacientes de unidades de terapia intensiva não tem suporte de dados clínicos e o controle da febre pode ser prejudicial, particularmente quando há uma doença infecciosa em evolução. Como este tópico é controverso, realizamos uma breve revisão sistemática e meta-análise da literatura para resumir as evidências atuais sobre o uso de antipiréticos em pacientes críticos.

## MÉTODOS

### Pesquisa da literatura e extração de dados

O banco de dados online MedLine (1966-2013) e o Registro Cochrane de Estudos Controlados (CENTRAL) foram pesquisados em busca de estudos que atendessem os seguintes critérios de inclusão: ensaios randomizados controlados; em pacientes críticos humanos adultos; em tratamento com antipiréticos em um braço x placebo ou não tratamento no outro; e relato de dados de mortalidade.

Os seguintes termos foram combinados na estratégia de busca, em inglês: *acetaminophen* [termo MeSH] (acetaminofeno) OU *NSAID* [termo MeSH] (AINES) OU *aspirin* [termo MeSH] (aspirina) OU *antipyrine* [termo MeSH] (antipirina) OU *cooling* (resfriamento) - E - *critical illness* [termo MeSH] (doença grave) OU *critical care* [termo MeSH] (cuidados críticos) OU *intensive care* [termo MeSH] (cuidados intensivos). Todos os artigos de revisão e estudos com referências cruzadas dos artigos selecionados foram triados em busca de informações pertinentes. Quando encontramos relatos em duplicidade do mesmo estudo em resumos preliminares e artigos, analisamos os dados relatados de modo mais completo.

### Análise dos dados e resultados

O desfecho primário foi mortalidade geral na UTI em pacientes tratados com antipiréticos em comparação aos não tratados. Os desfechos secundários incluíram alteração de temperatura, tempo de internação na UTI e tempo de internação hospitalar. De acordo com o critério PICOS, avaliamos: *P*, pacientes críticos; *I*, intervenção antipirética; *C*, sem tratamento antipirético; *O*, mortalidade na UTI; *S*, unidade de terapia intensiva.

Extraímos dados acerca do desenho do estudo, características dos pacientes, sobrevida global e alteração média na temperatura corporal. Para a análise de sobrevida, calculamos uma estimativa agrupada de razão de risco (RR) nos estudos individuais, através de um modelo de efeito aleatório segundo Mantel e Haenszel e representamos graficamente estes resultados em gráficos do tipo “*forest plot*”. Para variáveis contínuas, usamos a diferença média padronizada (SMD), que é a diferença em médias, dividida por um desvio padrão (DP). O pressuposto de homogeneidade foi verificado por uma teste χ^2^ com um *df* (graus de liberdade) igual ao número de estudos analisados menos 1 (um). A heterogeneidade foi também medida pelo I^2^, que descreve a porcentagem da variação total entre os estudos, que é devida à heterogeneidade mais que ao acaso. Calculou-se I^2^ a partir dos resultados básicos obtidos de uma meta-análise típica, pois I^2^ = 100% x (*Q* – df)/*Q*, onde *Q* é a heterogeneidade estatística de Cochrane e *df* são os graus de liberdade. A porcentagem de zero indica que não se observou heterogeneidade, e valores maiores mostram heterogeneidade crescente. Quando a heterogeneidade foi encontrada tentamos identificar e descrever a razão.

As variáveis paramétricas foram apresentadas como média±DP e as não paramétricas foram apresentadas como mediana (variação interquartil). Todas as análises foram realizadas com os programas *Revision Manager* v.5.1.1 e *Statistical Package for Social Sciences* (SPSS) v.16.0.1. Para todas as análises, os valores de p<0,05 foram considerados significantes.

## RESULTADOS

Uma busca abrangente da literatura rendeu 351 referências, das quais 339 artigos foram excluídos durante a primeira triagem, baseada em resumos ou títulos, deixando 12 artigos para revisão do texto completo. Durante essa revisão, nove artigos foram excluídos pelos seguintes motivos: estudo não randomizado (n=6); ambos grupos tratados (n=2); e nenhum dado sobre mortalidade (n=1). Finalmente, três artigos (320 participantes) foram incluídos na análise final^(3-5)^ (Figura 1 e Tabela 1).

**Figura 1:** Estratégia de pesquisa da literatura

**Tabela 1:** Qualidade científica dos estudos incluídos

Estudos	Ocultação da alocação	Semelhança no início do estudo	Interrupção precoce^*^	Perda de seguimento	Análise da intenção de tratar
Gozzoli et al.^(3)^ Pontuação Jadad: 3	Envelopes lacrados	Idade: semelhante Gravidade da doença: semelhante (SAPS II)	Não	Não	NS
Schulman et al.^(4)^ Pontuação Jadad: 3	Envelopes lacrados	Idade: semelhante Gravidade da doença: semelhante (APACHE II)	Não	Não	NS
Schortgen et al.^(5)^ Pontuação Jadad: 3	Sistema telefônico	Idade: semelhante Gravidade da doença: semelhante (SAPS III)	Não	Não	Sim

* : Interrupção precoce devido a benefício ou futilidade e presença de regras de interrupção explícitas *a priori*. NS: não significante.

Todos os três estudos analisados foram ensaios randomizados controlados e, em dois, o tratamento da febre foi com um dispositivo de resfriamento externo^(3,5)^ e, no último, com acetaminofeno.^(4)^ Em um estudo, os pacientes do grupo controle podiam ser tratados se a temperatura atingisse um determinado valor^(4)^ e, nos outros dois, nenhuma intervenção foi feita no grupo controle. Dois estudos avaliaram pacientes cirúrgicos^(3,4)^ e um avaliou pacientes em choque séptico durante internação na UTI.^(5)^ As características e os resultados dos estudos analisados estão expostos na tabela 2.

**Tabela 2:** Características e desfechos dos estudos incluídos na meta-análise

Características	Gozzoli et al.^(3)^		Schulman et al.^(4)^		Schortgen et al.^(5)^	
	Tratamento	Não tratamento	Tratamento	Não tratamento	Tratamento	Não tratamento
Cenário	Pacientes cirúrgicos		Pacientes cirúrgicos		Choque séptico	
Medição da T	Retal		Não definido		Temperatura central	
Número de pacientes	18	20	44	38	101	99
Idade, anos	54±13	53±19	47±20	47±20	62	61
Escores de gravidade	30^*^	28^*^	12.8^**^	11.4^**^	77^***^	79^***^
Início do tratamento antipirético	T≥38,5 + SIRS	Nunca	T>38,5	T>40,0	T>38,3	Nunca
Tipo de tratamento antipirético	Resfriamento externo	Nada	Acetaminofeno	Acetaminofeno	Resfriamento externo	Nada
Objetivo do tratamento antipirético	T≤37.5	Nada	T<38.5	T<40.0	T<37.0	Nada
T inicial, ºC	38.9±0.3	38.8±0.5	38.3±0.8	38.3±0.7	38.8±0.8	38.9±0.7
T após 24 horas, ºC	37.6±0.5	37.7±0.6	36.6±0.6	37.7±0.5	36.7±0.6	38.1±0.5
Tempo de internação na UTI, dias	11±13	9±10	22±30	20±14	17±14	16±17
Tempo de internação, dias	28±22	31±24	-	-	36±40	28±31
Mortalidade na UTI, n (%)	2 (11)	3 (15)	7 (16)	1 (3)	35 (35)	43 (43)

* : SAPS II (*Simplified Acute Physiology Score*) (Pontuação Fisiológica Aguda Simplificada);

** : APACHE II (*Acute Physiology and Chronic Health Evaluation*) II (Fisiologia Aguda e Avaliação de Saúde Crônica);

*** : SAPS III. T: temperatura; SIRS: síndrome da resposta inflamatória sistêmica; UTI: unidade de terapia intensiva.

Dos 163 pacientes, 44 (27%) do grupo de tratamento da febre, e 47 de 157 pacientes (30%) do grupo controle morreram durante a internação na UTI (RR: 0,91; intervalo de confiança de 95% – IC 95%: 0,65-1,28). Não houve diferença na análise estratificada entre pacientes cirúrgicos e pacientes com choque séptico (RR: 2,19; IC 95%: 0,68-7,06; e RR: 0,80; IC 95%: 0,56-1,13, respectivamente). Houve uma leve heterogeneidade entre os resultados (Tabela 3 e Figura 2). A inspeção visual do gráfico de funil para análise de sobrevida revelou simetria e o teste de Begg não foi estatisticamente significativo (p=0,54).

**Tabela 3:** Características e desfechos dos pacientes analisados na meta-análise

	Tratamento (n=163)	Controle (n=157)	Valor de p	SMD/RR (95%IC)	Heterogeneidade	Valor de p
Idade, anos	54,33±7,59	53,66±7,02	0,916	-	-	-
Temperatura inicial, ºC	38,66±0,32	38,40±0,45	0,456	-0,06 (-0,27-0,16)	0,560	0,620
Temperatura após 24 horas, ºC	36,96±0,55	37,83±0,23	0,066	-1,57 (-2,86- -0,29)	<0,0001	0,020
Mudança da temperatura, ºC	-1,70±0,40	-0,56±0,25	0,014	-	-	-
Internação na UTI, dias	16,66±5,50	15,00±5,56	0,731	0,08 (-0,14-0,30)	0,960	0,470
Internação hospitalar, dias	32,00±5,65	29,50±2,12	0,618	0,17 (-0,09-0,42)	0,320	0,200
Mortalidade na UTI, número (%)	44 (27)	47 (30)	0,637	1,13 (0,40-3,15)	0,140	0,820

SMD: diferença media padronizada; RR: razão de risco; IC 95%: intervalo de confiança de 95%; UTI: unidade de terapia intensiva. IC 95%: intervalo de confiança de 95%.

**Figura 2:** Meta-análise da sobrevida global de pacientes críticos com ou sem tratamento antipirético

Não há diferença no tempo de internação na UTI e no hospital entre pacientes tratados e controles (Tabela 2 e Figura 3). Como esperado, os pacientes tratados com tratamento antipirético tiveram maior redução na temperatura em 24 horas e menor temperatura corporal ao final do seguimento (Tabela 2 e Figuras 4 e 5).

**Figura 3:** Meta-análise do tempo de internação de pacientes críticos em unidade de terapia intensiva e no hospital, com ou sem tratamento antipirético

**Figura 4:** Meta-análise da temperatura inicial e final de pacientes críticos após 24 horas com ou sem tratamento antipirético

**Figura 5:** Mudança de temperatura após 24 horas em pacientes tratados com antipiréticos (círculo preto e linha contínua) e controles (quadrado preto e linha tracejada)

## DISCUSSÃO

Encontramos evidências de que o uso de tratamento antipirético para controle da febre em pacientes críticos não estava associado a melhores desfechos em comparação a pacientes não tratados. Chama atenção o fato de a redução na temperatura durante as primeiras 24 horas, e na temperatura após 24 horas terem sido significativamente menores no grupo tratado com antipiréticos.

A febre é reconhecida como um sinal de doença há 4.500 ou 5.000 anos.^(6)^ Deve-se a várias moléculas endógenas capazes de modificar a temperatura normal. Apesar de a atividade da pirexina ser possivelmente devida a uma contaminação por endotoxina, a substância que produz febre, a partir dos leucócitos polimorfonucleares, e o pirogênio endógeno eram candidatos e agora são reconhecidos como citocinas pirogênicas.^(6-9)^

Na década de 1970, Kluger et al.^(2)^ mostraram que lagartos infectados com bactérias a 42°C permitiam que elas sobrevivessem, enquanto que todos morriam quando mantidos a 34°C. Trinta anos mais tarde, Jiang et al.^(10)^ realizaram um experimento semelhante com camundongos. Uma infecção peritoneal foi inoculada em camundongos mantidos em um ambiente que permitia uma temperatura central de 37,5 ou 39.7°C. A carga bacteriana foi exponencial na cavidade peritoneal de camundongos sem febre e ficou sob controle nos camundongos com febre. Todos os camundongos sem febre morreram enquanto que 50% dos animais com febre sobreviveram.

Descobriu-se que a febre está associada a melhores desfechos em humanos em vários estudos observacionais. Em pacientes com bacteremia por Gram-negativo, a febre foi um dos fatores associados a menor mortalidade.^(11)^ Em pacientes idosos com pneumonia adquirida na comunidade, febre e leucocitose também estiveram associadas a menor mortalidade.^(12)^ Devido a numerosos modelos animais experimentais de infecção grave demonstrando que o tratamento antipirético aumenta a mortalidade, a comunidade médica foi alertada sobre o uso de tratamento antipirético em pacientes sépticos.^(13)^

Recentemente, Lee et al.^(1)^ mostraram que, em pacientes críticos, a relação entre febre e mortalidade variava de acordo com a intensidade da febre e estava independentemente associada a mortalidade apenas no subgrupo ≥39,5°C de pacientes sem sepse. Neste grupo de pacientes, pode-se pressupor que a febre alta é provavelmente causada por infecção e isso pode explicar a mortalidade. Febre alta está associada a arritmias cardíacas, aumento da demanda por oxigênio, dano cerebral e convulsões.^(14,15) ^Em pacientes com febre não infecciosa, estes efeitos prejudiciais ocorrerão sem o benefício potencial da proteção relacionada a febre.^(1)^

Imagina-se que a febre iniba a atividade de vírus e bactérias e o tratamento antipirético pode reduzir esta ação.^(1,16)^ Além disso, o tratamento antipirético em pacientes sépticos com antiinflamatórios não esteroidais e acetaminofeno pode ser tóxico, pois pode estar associado à hipotensão e disfunção renal.^(17)^ Novamente, Lee et al.^(1)^ mostraram que a mortalidade é mais alta entre pacientes sépticos que não conseguem desenvolver febre, corroborando o argumento de que a febre pode ser naturalmente protetora. Um estudo de pacientes que sofreram traumatismo foi interrompido prematuramente devido à tendência a aumento no risco de infecção e óbito em pacientes tratados agressivamente com acetaminofeno e resfriamento físico.^(4)^ Além disso, dois estudos referiram que a terapia com ibuprofeno em pacientes com sepse não influenciou na mortalidade.^(18,19)^

As limitações de nosso estudo incluem o risco de viés, que pode exagerar a conclusão do estudo se a publicação estiver relacionada a força dos resultados. Além disso, apenas três artigos foram incluídos, o que aumenta o viés destes estudos. Pesquisamos referências em poucos bancos de dados e usamos uma estratégia de pesquisa simples, o que poderia levar à perda de alguns estudos. A análise de resfriamento físico juntamente com fármacos poderia ser outra fonte de viés.

Um grande estudo randomizado controlado está sendo realizado para confirmar o real efeito do tratamento antipirético em pacientes críticos.^(20)^

## CONCLUSÃO

Os resultados da presente revisão sugerem que o tratamento antipirético em pacientes críticos não está associado a melhor sobrevida se comparado a nenhum tratamento para febre. Outros estudos com mais dados são necessários para confirmar o efeito do controle da febre sobre a mortalidade e para determinar se uma leve hipotermia traz benéficos adicionais a pacientes críticos
